# *CMJ*’s twentieth anniversary: excellence for the future

**DOI:** 10.3325/cmj.2011.52.443

**Published:** 2011-08

**Authors:** Ana Marušić, Ivan Damjanov

*Whereof what's past is prologue; what to come, In yours and my discharge.* Shakespeare The Tempest, Act 2, scene 1, 253-254

On the twentieth anniversary of its first publication, the *Croatian Medical Journal* (*CMJ*) is ready for a new future. The team that made the *Journal* what it is today is leaving – its two Co-editors in Chief and almost the whole Editorial Board. The two Co-editors in Chief have been elected as *editors emeriti*, the title that had already been given to the founder and creator of the *CMJ*, Prof. Matko Marušić, who stepped down as the editor when he became the dean of the School of Medicine at the University of Split (1).

We are leaving because we thought that a twentieth anniversary was an appropriate time for the *CMJ* to rethink its role in the scientific community and to make a new strategy for its development. It was easy for us to leave as we were not bound to the Journal by financial benefits – Editorial Board membership and the positions of Editors in Chief have always been voluntary. This gave us a lot of freedom and some troubles but, most of all, great creativity in our passion to run a journal opened to the small and developing scientific communities (2-20).

Over the first 20 years (and we count from the first publication of the *CMJ* – its first War Supplement published before the first official issue in 1992), we have probably reached the peak of achievement for a small general medical journal from a small country. We always closely followed the journal in its childhood and adolescent years (21-25) and were good in predicting the impact of the *Journal* in the mainstream science. On *CMJ*’s fifteenth birthday in 2006, we were about to get our first official impact factor and predicted that it would be over 0.8 (24). It was 0.825 and it steadily increased over the years to 1.455 for 2010, as released by Thomson Reuters’ Journal Citation Reports at the end of June 2011 (26). This was also the highest impact factor any scholarly journal in Croatia ever received.

In 2006, we also reaffirmed our position that the goal of the *CMJ* was not to increase its impact factor but to increase its activities as a busy two-direction bridge between the small and large scientific communities. Instead of racing for the impact factor, we opened “new doors and windows into areas such as research integrity, education in scientific writing, and excellence in publishing clinical research” (24).

With this editorial we do not say goodbye to our colleague editors and authors – all of us will go back to our busy academic lives and hopefully make up for our absence. As a picture is worth a thousand words, we tell the history of the *CMJ* with all of its cover pages up to this issue (Figure 1). In words, we rather set the stage for the next twenty years of *CMJ*’s adult life.

**Figure 1:**
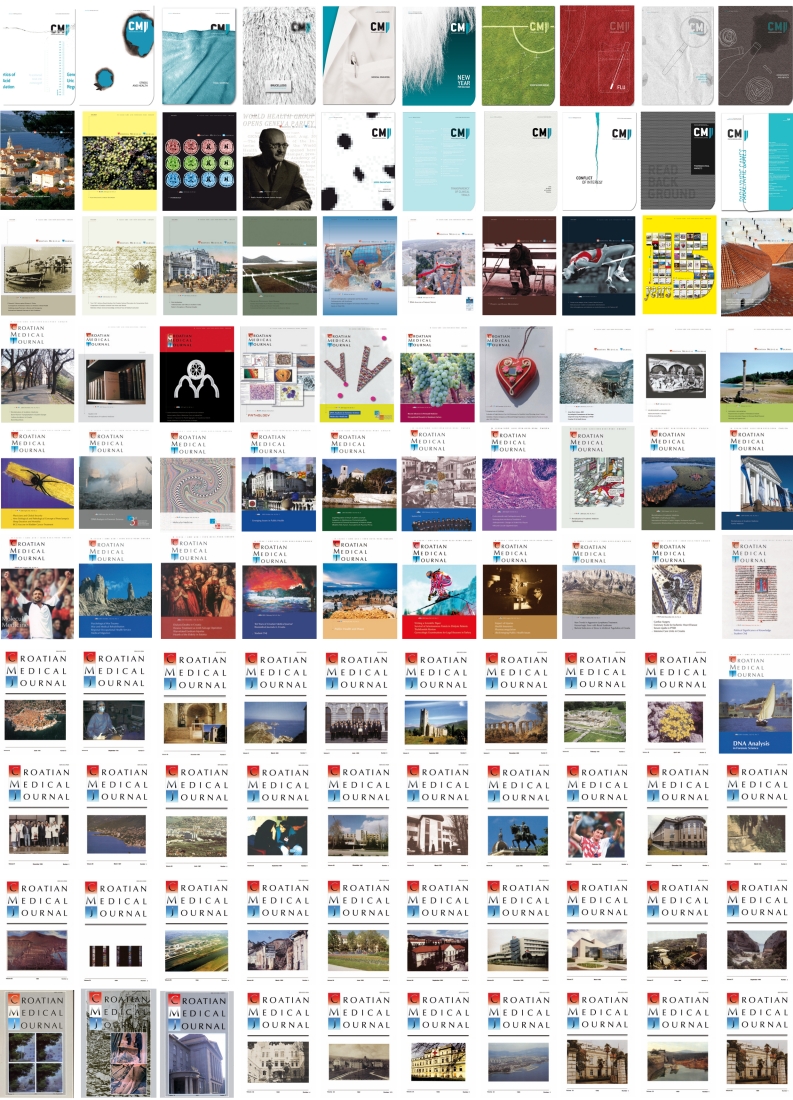
History of the Croatian Medical Journal in its cover pages, 1991-2011.

Our strategy to develop the *CMJ* as the door for the small scientific communities to the mainstream science and the window for the world to see good research in small and developing countries (22-25) has been accomplished. More than that, we have created a niche for the journal by focusing on specific topics, from war and peace related research, over public health issues to, most recently, population genetics and forensic DNA research (27).

We believe that the latter topics are the future of the *CMJ* – to become a publishing outlet for the wealth of research in molecular medicine around the world. Statistics of the cross-citations between the *CMJ* and other journals provides solid evidence base for this proposal. As in previous years, the relatedness statistics (28) of the 2010 Journal Citation Reports show that the first three journals most related to the *CMJ* are forensic science journals*: Forensic Science International: Genetics*, *International Journal of Legal Medicine*, and *Forensic Science International*. Since 2001, the *CMJ* has been publishing a theme issue dedicated to forensic DNA research every two years, and the articles have always been our most-cited publications (25). In 2006, forensic theme issues were joined by theme issues on population genetics and its relevance for health and disease in the global human population (29).

Now that we have left (30), we hope that our colleagues from the new editorial team will take the *CMJ* further along this exciting and challenging path. It is better that the past does not restrain them. All advice we can give was already voiced many years ago by Mark Twain (31):

“Twenty years from now you will be more disappointed by the things that you didn't do than by the ones you did do. So throw off the bowlines. Sail away from the safe harbor. Catch the trade winds in your sails. Explore. Dream. Discover.”

## References

[1] Marusic M (2009). Conflict of interest for editor: sweet and sad choices. Croat Med J.

[2] Marusic A, Marusic M (2003). Teaching students how to read and write science: a mandatory course on scientific research and communication in medicine.. Acad Med.

[3] Hren D, Vujaklija A, Ivanisevic R, Knezevic J, Marusic M, Marusic A (2006). Students' moral reasoning, Machiavellianism and socially desirable responding: implications for teaching ethics and research integrity.. Med Educ.

[4] Hren D, Lukic IK, Marusic A, Vodopivec I, Vujaklija A, Hrabak M (2004). Teaching research methodology in medical schools: students' attitudes towards and knowledge about science.. Med Educ.

[5] Burazeri G, Civljak M, Ilakovac V, Jankovic S, Majica-Kovacevic T, Nedera O (2005). Survey of attitudes and knowledge about science in medical students in southeast Europe.. BMJ.

[6] Godlee F, Marusic M. Re: journals are also responsible. Rapid response to Chalmers I, BMJ. 2006;333:594-595. Available from: http://www.bmj.com/cgi/eletters/333/7568/594 Accessed: July 15, 2011.

[7] Puljak L (2007). Croatia founded a national body for ethics in science.. Sci Eng Ethics.

[8] Godlee F (2007). Plagiarism and punishment.. BMJ.

[9] Kmietowicz Z (2007). University drops case against Croatian academic accused of plagiarism.. BMJ.

[10] Watts G (2007). Croatian academic is found guilty of plagiarism.. BMJ.

[11] Marusic A, Katavic V, Marusic M (2007). Role of editors and journals in detecting and preventing scientific misconduct: strengths, weaknesses, opportunities, and threats.. Med Law.

[12] Vogel G (2008). Croatian editors fight with Medical school over journal's fate.. Science.

[13] Cikes N (2008). The case against the CMJ's editors.. Science.

[14] Marusic M, Marusic A (2008). The future of the CMJ.. Science.

[15] (2008). Corrections and clarifications.. Science.

[16] Marcovitch H. Croatian Minister replies but medical school silent. Rapid response for Markovitch. BMJ. 2008;336:174. Available from: http://www.bmj.com/cgi/eletters/336/7637/174 Accessed: July 15, 2011.

[17] Marcovitch H (2008). Croatia is let down.. BMJ.

[18] Sibbald B, Flegel K (2008). Integrity at the Croatian Medical Journal.. CMAJ.

[19] Vlassov VV (2008). Dangers of doing right thing in a wrong place.. Eur J Public Health.

[20] Vujaklija A, Hren D, Sambunjak D, Vodopivec I, Ivanis A, Marusic A (2010). Can teaching research methodology influence students' attitude toward science? Cohort study and nonrandomized trial in a single medical school.. J Investig Med.

[21] Lackovic Z (1992). Who needs Croatian Medical Journal?. Croat Med J.

[22] Marusic M (1997). Life of an Editor in Chief: First five years.. Croat Med J.

[23] Marusic A, Misak A, Kljakovic-Gaspic M, Marusic M (2002). Educatione ad excelentiam – ten years of the Croatian Medical Journal.. Croat Med J.

[24] Marusic M, Sambunjak D, Marusic A (2006). Life of small medical journal-how bibliographical indexing and international visibility affected editorial work in Croatian Medical Journal.. Croat Med J.

[25] Kovacic N, Huic M, Ivanis A (2008). Citation analysis of the Croatian Medical Journal: the first 15 years.. Croat Med J.

[26] Reuters T. Thomson Reuters Releases Journal Citation Reports for 2010. 28 June 2011. Available from: http://thomsonreuters.com/content/press_room/science/JCR-impact-factor-2010 Accessed: July 15, 2011.

[27] Marusic A (2009). Morphology: bodies, genes, journals.. Croat Med J.

[28] Pudovkin A, Garfield E (2002). Algorithmic procedure for finding semantically related journals.. J Am Soc Inf Sci Technol.

[29] Rudan I (2006). The land of 1000 islands.. Croat Med J.

[30] Rudan I, Marusic A, Campbell H (2011). Developing biobanks in developing countries. Journal of Global Health..

[31] Twain M. Quotes.net. STANDS4 LLC, 2011. 15 July. 2011. Available from: http://www.quotes.net/quote/1681 Accessed: July 15, 2011.

